# Beclin-1–p53 interaction is crucial for cell fate determination in embryonal carcinoma cells

**DOI:** 10.1111/jcmm.12386

**Published:** 2014-09-11

**Authors:** Rakshamani Tripathi, Dipankar Ash, Chandrima Shaha

**Affiliations:** Cell Death and Differentiation Research, National Institute of ImmunologyNew Delhi, India

**Keywords:** embryonal carcinoma cells, cisplatin, apoptosis, autophagy, p53, Beclin-1

## Abstract

Emerging interest on the interrelationship between the apoptotic and autophagy pathways in the context of cancer chemotherapy is providing exciting discoveries. Complexes formed between molecules from both pathways present potential targets for chemotherapeutics design as disruption of such complexes could alter cell survival. This study demonstrates an important role of Beclin-1 and p53 interaction in cell fate decision of human embryonal carcinoma cells. The findings provide evidence for p53 interaction with Beclin-1 through the BH3 domain of the latter. This interaction facilitated Beclin-1 ubiquitination through lysine 48 linkage, resulting in proteasome-mediated degradation, consequently maintaining a certain constitutive level of Beclin-1. Disruption of Beclin-1–p53 interaction through shRNA-mediated down-regulation of p53 reduced Beclin-1 ubiquitination suggesting requirement of p53 for the process. Reduction of ubiquitination consequently resulted in an increase in Beclin-1 levels with cells showing high autophagic activity. Enforced overexpression of p53 in the p53 down-regulated cells restored ubiquitination of Beclin-1 reducing its level and lowering autophagic activity. The Beclin-1–p53 interaction was also disrupted by exposure to cisplatin-induced stress resulting in higher level of Beclin-1 because of lesser ubiquitination. This higher concentration of Beclin-1 increased autophagy and offered protection to the cells from cisplatin-induced death. Inhibition of autophagy by either pharmacological or genetic means during cisplatin exposure increased apoptotic death *in vitro* as well as in xenograft tumours grown *in vivo* confirming the protective nature of autophagy. Therefore, Beclin-1–p53 interaction defines one additional molecular subroutine crucial for cell fate decisions in embryonal carcinoma cells.

## Introduction

The study of cross talk between the processes of apoptosis and autophagy has attracted substantial attention in recent years because of the awareness that manipulation of these processes can potentially improve chemotherapeutic efficacy 1. While apoptosis is a cell death process also able to serve as a survival mechanism within a group of cells like a tumour, autophagy is a conserved lysosomal degradation pathway helping cell survival or inducing death 2. This apparently paradoxical effect of the two processes and their intimate relationship in determining cell fate requires an in-depth understanding of their connections. Two primary molecules with influence on both apoptosis and autophagy are the tumour suppressors Beclin-1 and p53 3. While p53 is known to be defective in 50% of human cancers 4, Beclin-1, with a primary role in autophagy, is monoallelically deleted in human breast and ovarian cancers 5. Beclin-1 and vacuolar sorting protein 34 (Vps34), a class-III phosphoinositide 3-kinase (PI3K), bind to each other and initiate the formation of autophagosome 6. Unlike Beclin-1, p53 plays a primary role in limiting oncogenesis through cell cycle arrest for repair or by inducing apoptosis 7,8. The p53 binds to members of the Bcl-2 family to mediate transcription-independent apoptosis 9,10. Under various conditions, autophagy can be regulated by p53 either for a protective or a death-inducing role through the induction of multiple proteins 3.

Beclin-1 contains a short BH3 motif through which it binds to Bcl-2 11 and this interaction is responsible for tuning the Vps34 activity 12 leading to modulation of autophagy under various conditions 13. Disruption of this binding forms the basis of development of inhibitory compounds, several of which are undergoing clinical trials for the treatment of cancer 14. New drugs, like a BH3 mimetic that targets interaction of Bcl-2 family members within themselves or with other proteins, show promise in treatment of several types of cancer 15. Therefore, protein–protein interactions are important in determining cell fate and such interactions often regulate a variety of cellular events including ubiquitination 16. Prior knowledge suggests that both Beclin-1 and p53 levels are regulated through the process of ubiquitination 17,18. Ubiquitination of Beclin-1 regulates function of the protein through proteolytic or non-proteolytic action 18 indicating the importance of the process.

Embryonal carcinomas (EC) are germ cell tumours that are relatively common among testicular germ cell tumours and occur more frequently in the testis than in the ovary 19. As they occur mostly in young patients, aggressive therapy is usually given 19. The tumours formed by EC cells, although initially sensitive to drugs, acquire resistance during therapy 20. Although a sizeable amount of work has been carried out with EC cells, how autophagy and apoptosis are regulated in these cells is not clear. Studies are required to illuminate these aspects, so as manipulation of cellular processes during therapy may help improve treatment efficacy. Our earlier studies with EC cells demonstrated that p53 down-regulation was protective to the chemotherapeutic drug cisplatin 21. In this study, we show a new observation that the protective action of p53 lowering was because of increased autophagy as a result of Beclin-1 elevation caused by reduced ubiquitination because of disruption of p53–Beclin-1 interaction.

## Materials and methods

### Reagents and antibodies

Foetal calf serum (FCS) and Embryonic Stem Cell grade FCS (ES-FCS) were procured from Biological Industries (Kibbutz Beit Haemek, Israel). Trypsin-EDTA was obtained from Himedia (Mumbai, India). CB-X™ protein assay kit and femto LUCENT™ PLUS-HRP Chemiluminescent reagent kit were purchased from G-Biosciences^©^ (St. Louis, MO, USA). Antibodies to Beclin-1 and Matrigel™ were from BD Biosciences (San Jose, CA, USA). Antibodies against PARP, GFP and p53 were from Santa Cruz Biotechnology (Santa Cruz, CA, USA) and antibodies against FLAG® M2, LC3B, ATG-5, Beclin-1 (D40C5), Beclin-1 (2A4), p62 (D5E2), cleaved caspase-3, K48-linkage specific polyubiquitin and protein A and protein G magnetic beads were from Cell Signaling Technology (Danvers, MA, USA). Anti-tubulin and anti-pan β-actin antibody came from NeoMarkers (Fremont, CA, USA). Antibody against HA epitope was obtained from NOVAS Biologicals (Littleton, CO, USA). Secondary Rabbit TrueBlot®: antirabbit IgG HRP and Mouse TrueBlot®: antimouse IgG HRP was obtained from eBioscience (San Diego, CA, USA). Secondary antimouse and antirabbit antibodies conjugated to horseradish peroxidase were obtained from Jackson Immuno Research Laboratories Inc. (West Grove, PA, USA). Normal Goat Serum was procured from GIBCO BRL (Gaithersburg, MD, USA). The shRNAs against Beclin-1, ATG5, vector control and scrambled shRNA were obtained from ORIGENE (Rockville, MD, USA). DMEM (with and without phenol red), RIPA buffer, cisplatin (cis-diamminedichloro platinum (II)), propidium iodide (PI), chloroquine, bafilomycin A1, 3-MA (3-methyladenine), Wortmannin, ethylenediamine-tetraacetic acid, MG132 were obtained from Sigma-Aldrich (St. Louis, MO, USA). Protease cocktail inhibitors were purchased from Roche (Indianapolis, IN, USA).

### Cell lines and culture

The NT2/D1 cells, a pluripotent cell line (kind gift from Dr. M. Inamdar of JNCASR, Bangalore with consent from Dr. P. Andrews, University of Sheffield, UK) derived from a human teratocarcinoma, were maintained in DMEM with 10% heat-inactivated ES grade FCS at 37°C in a humidified atmosphere of 95% air and 5% CO_2_. Cells were characterized for specific markers as reported earlier 21.

### Death assays and immunohistochemistry

Annexin-V/PI and TUNEL staining: The Vybrant apoptosis assay kit was used to perform Annexin-V/PI staining as described previously 21. For TUNEL staining, DeadEnd Fluorometric TUNEL system was used where cells were fixed with 4% paraformaldehyde (methanol-free) for 10 min. at room temp and subsequently permeabilized in 0.2% Triton X100. Recombinant terminal deoxynucleotidyl transferase (TdT) and fluorescein-12-dUTP were used to label the cells for 2 hrs at 37°C in dark. Reaction was terminated with 20 mM EDTA. Results were analysed by using FACS Calibur (BD Biosciences, CA, USA). All immunochemical procedures were carried out as described previously 21.

### Co-immunoprecipitation

Cells were lysed with non-denaturing lysis buffer (20 mM Tris HCl pH 8.0, 137 mM NaCl, 10% glycerol, 1% Nonidet P-40, 2 mM EDTA) and protease inhibitor cocktail (Roche). After centrifugation for 30 min. at 4°C, supernatants were pre-cleared with protein A/G beads to avoid non-specific binding during immunoprecipitation. After pre-clearing, a fraction was saved as input. Pre-cleared lysates were incubated for 1 hr with antibody cross-linked to protein A/G beads. After multiple washes with lysis buffer, beads were dried and bound proteins were eluted in 2× Laemmli buffer. The samples were boiled at 95°C for 5 min., centrifuged and separated by SDS-PAGE 21.

### Ubiquitination assay

For detection of ubiquitinated proteins, cells were transfected with 5 μg of 6× His-ubiquitin expression plasmid along with equal amounts of various Beclin-1-expressing plasmids. To equalize the DNA amount, pcDNA3 vector was used. After 36 hrs of transfection, 5 μM MG132, the proteasome inhibitor was added along with cisplatin and cells were further incubated for 12 hrs. After treatment, cells were suspended in 1 ml lysis buffer (6 M guanidinium-HCl, 0.1 M Na_2_HPO_4_/NaH_2_PO_4_ and 10 mM imidazole), sonicated and centrifuged. Ni-NTA beads were added to the supernatant for 4 hrs at room temp. Subsequently, after one wash with lysis buffer [1X lysis buffer diluted in 25 mM TrisHCl (pH 6.8) (1:4) 20 mM imidazole] and two washes with another buffer (25 mM TrisHCl, pH 6.8, fortified with 20 mM imidazole), ubiquitinated proteins were eluted from the beads. The beads were incubated with sample loading buffer containing 200 mM imidazole. The eluted proteins were separated on 8–10% SDS-PAGE followed by immunoblotting with anti-p53 and anti-Beclin-1 antibodies. For *in vivo* ubiquitination assay, cells were transiently cotransfected with GFP p53 and ubiquitin expression (HA-Ub) vectors. After 24–36 hrs of transfection, cells were cultured with or without proteasome inhibitors for 12–16 hrs. Cells were lysed in RIPA buffer containing protease inhibitor cocktail and 10 μM MG132. The lysates were diluted to a solution with IP buffer and immunoprecipitations were carried out with anti-Beclin-1 antibody. The ubiquitinated proteins were separated by SDS-PAGE and analysed by western blot by using anti-HA and anti-ubiquitin antibody.

### SDS-PAGE and Western Blot

SDS-PAGE and western blots were carried out as described previously 21. Dilutions for different antibodies used for western blots were as follows: anti-caspase-8, anti-caspase-3, anti-caspase-9, anti-LC3B, anti-ap62, anti-ATG5, anti-Beclin-1, anti-HA, anti-ubiquitin (1:1000), anti-GFP, anti-p53, anti-PARP (1:4000), anti-tubulin and anti-actin (1:10,000) in PBS-Tween 20 containing 1–5% of appropriate blocking reagent.

### Transfections

DNA and Lipofectamine LTX plus were diluted in serum-free OPTI-MEM and incubated for 5 min. at room temp. Subsequently, the DNA and Lipofectamine dilutions were combined and incubated for 30 min. at room temp and Lipofectamine-DNA complexes were added to cells. The reaction was stopped after 5–8 hrs with fully supplemented DMEM medium.

### Lentivirus-mediated RNA interference

Cells were transduced with lentivirus carrying shRNA designed to knock down p53 (Addgene plasmid 19119) or scramble shRNA (Addgene plasmid 1864) as described previously 21.

### Nuclear and cytosolic fractionation

Nuclear–cytoplasmic fractionation was carried by using the NE-PER Nuclear and Cytoplasmic Extraction Reagents kit (Pierce Biotechnology, Rockford, IL, USA) according to the manufacturer’s protocol. Protease inhibitor tablets (Roche Diagnostics, GmbH) were added to the CERI and NER extraction reagents prior to use. Immunoprecipitation experiments were performed from cytoplasmic and nuclear fractions by using p53 and Beclin-1 as immunoprecipitating antibodies.

### Quantification of number of GFP-LC3 puncta

GFP-LC3 puncta were counted from cells transfected with GFP-LC3 and subsequently treated with or without cisplatin and other agents. Images captured at 40X magnification with Leica TCS SP5 II (Leica Microsystems, Wetzlar, Germany) confocal microscope were processed for algorithmic quantification of GFP-LC3 puncta per cell by using custom-written Image J macro-containing plug-ins as described by Chu *et al*. 22. At least 50 cells per sample were counted.

### Drug administration in mouse allografts

Tumour assays were performed with Institutional Animal Ethics Committee (National Institute of Immunology, New Delhi, India) approved protocols. Cells (10^6^ cells/injection) were inoculated subcutaneously to form tumours. Mice with tumours (300 mm^3^) were divided into four groups (6 mice/group). Vehicle or cisplatin (2 mg/kg/day, 3 days/week, 3 cycles) or wortmannin (0.4 mg/kg/day, 3 days/week, 3 cycles) alone or in combination was administrated intraperitoneally. Tumour volume was measured by using Vernier callipers (major and minor axis) and calculated by the equation: L × W^2^/2 (mm^3^), where L = length and W = width.

### Statistical analysis

Data are reported as mean ± SEM unless mentioned. Comparisons were made between different treatments by using the unpaired Student’s *t*-test and Mann–Whitney rank sum test. Differences were considered significant at *P* < 0.05 for both tests.

## Results

### Down-regulation of p53 increases cellular autophagy

**Figure 1:**
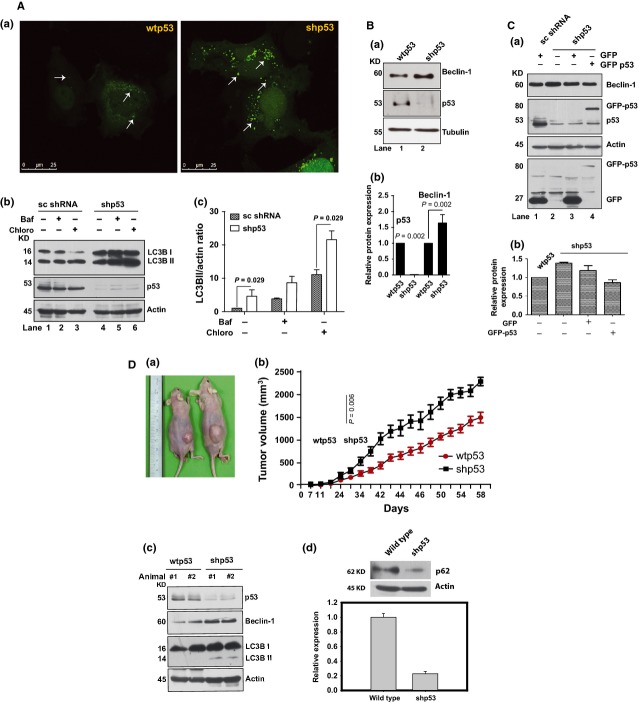
Knockdown of p53 enhances Beclin-1 expression and autophagy. (**A**, **a**) The wtp53 and shp53 cells transfected with GFP-LC3 show GFP-LC3 puncta. Arrows indicate autophagosomal vesicles. (**b**) Western blot shows expression of LC3BII in sc shRNA and p53 shRNA transfected cells treated with bafilomycin A1 (20 nM) or 100 μM chloroquine for 3 hrs. Chloro, chloroquine; Baf, bafilomycin A1. (**c**) Bar graph shows LC3BII/actin ratio on western blots shown in (**b**) quantified by densitometric analysis. *P*-values indicate Mann–Whitney rank sum test significance. (**B**, **a**) Increase in Beclin-1(lane 2) in p53 down-regulated cells as compared with wtp53 cells (lane 1). (**b**) Bar graph showing ratio of Beclin-1/actin and p53/actin of western blot shown in (**a**). Data are mean ± SEM (*n* = 3), *P*-values indicate Mann–Whitney rank sum test significance. (**C**, **a**) Western blots of Beclin-1, p53, GFP-p53 and GFP in shp53 cells transfected with indicated plasmid. sc shRNA cells were transfected with GFP plasmid only. Actin was used as a loading control. All western blots are representative of a minimum of 3–4 repeats. (**b**) Bar graph showing the results of densitometry, note showing lower protein amounts in lane 4. (**D**, **a**) Representative photograph of tumours formed by wtp53 and shp53 cells in the right flanks of *nu/nu* mice (*n* = 6). (**b**). Graph shows tumour volume changes over days. (**c**) Western blots of tumour lysates obtained from wtp53 and shp53 primary xenografts, probed with primary antibodies to p53, LC3BII and Beclin-1 with actin as loading controls. (**d**) Western blots of tumour lysates generated by wtp53 and shp53 cells probed with anti-p62 antibody. Bar graph shows the densitometry. All western blots are representative of a minimum of 3–4 repeats.

Based on our earlier study showing an increase in EC cell survival upon down-regulation of p53 21, we sought to understand the mechanism of this process by using EC cells with compromised levels of p53 (shp53). A significant p53 down-regulation was achieved through transfection with shRNA against p53 mRNA (Fig. S1). For estimation of autophagic activity, the shp53 cells were transfected with GFP-LC3. LC3, a soluble protein present in the cytosol, forms LC3-phosphatidylethanolamine (LC3BII) during autophagy when conjugated to phosphatidylethanolamine, which is recruited to autophagosomal membranes 23. Linked to GFP, LC3 puncta formation can be used as a marker of autophagy under the microscope. A large number of autophagic vacuoles were observed in shp53 cells as compared with the wild-type (wtp53) cells (Fig. 1A, a). As autophagy is a continuous process in normal cells, for actual detection of increased autophagy, chloroquine and bafilomycin A1 the two autophagy inhibitors were used for evaluation of autophagy flux. Chloroquine inhibits autophagy by endosomal acidification, thus preventing activation of lysosomal enzymes and bafilomycin A1 inhibits the fusion between autophagosomes and lysosomes 24. The detection of high levels of LC3BII protein (Fig. 1A, b, lanes 5, 6 & c) in the presence of the two inhibitors confirmed the microscopic evidence of increased formation of autophagic vacuoles in shp53 cells as compared with cells transfected with scrambled (sc) shRNA only.

### Beclin-1 increases under p53 down-regulated conditions

The above data of increased autophagy upon down-regulation of p53 prompted us to check Beclin-1 levels, a protein with a crucial role in autophagy 5. In shp53 cells, Beclin-1 was significantly higher (Fig. 1B, a, lane 2; b) than in wtp53 cells (Fig. 1B, a, lane 1; b) relating high Beclin-1 to increased autophagy as reported in many studies 6. Arguably, if p53 lowering was causal to Beclin-1 increase, restoring the p53 levels in shp53 cells should lower Beclin-1. Supporting this idea, shp53 cells compensated for p53 through transfection with GFP-p53 overexpression plasmids showed lower Beclin-1 levels (Fig. 1C, a, lane 4) as compared with cells transfected with sc shRNA, untransfected cells or only GFP transfected cells (Fig. 1C, a, lane 1–3). However, it appears that transfection with GFP-only plasmid lowers Beclin-1 levels in lanes 1 and 3, but comparatively, the level of Beclin-1 was lowest in lane 4 where GFP p53 was expressed (Fig. 1C, b).

The shp53 and wtp53 cells were used to generate xenograft tumours in *nu/nu* mice where shp53 cells showed much faster and comparatively larger formation of tumours (Fig. 1D, a). Figure 1D, b shows the relative increase in volume of tumours generated by wtp53 and shp53 cells. Tumour lysates from xenograft tumours showed higher Beclin-1 levels in shp53 tumours (Fig. 1D, c, lanes 3, 4) as compared with lysates from wtp53-cell induced tumours (Fig. 1D, c, lanes 1, 2). The above data were supplemented by the observation of lower p62 levels in shp53-cell generated tumours as decrease in p62 levels is indicative of increased autophagy 25. Immunohistochemistry with tumour sections showed high Beclin-1 and LC3BII in tumours formed by shp53 cells, whereas, tumours formed by wtp53 cells showed lower LC3BII and Beclin-1 (data on request). This corroborated the *in vitro* data and clearly established that lowering of p53 favours increase in Beclin-1 with increased autophagy both *in vitro* and *in vivo*.

### p53 binds to Beclin-1

**Figure 2:**
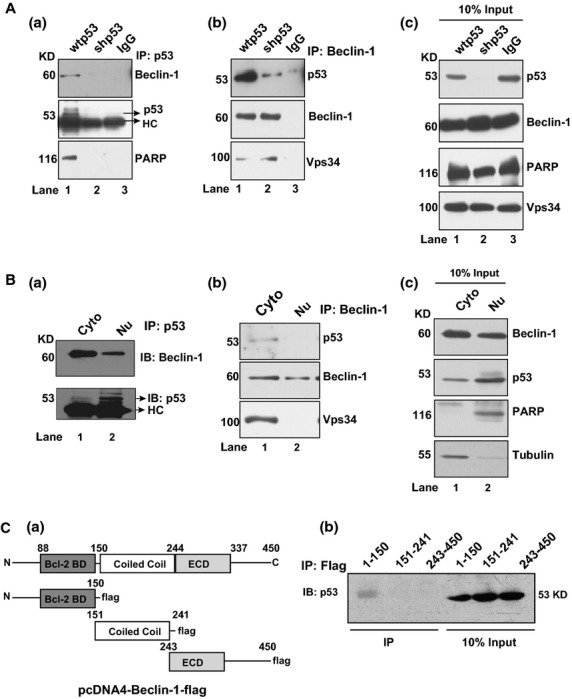
Endogenous p53 and Beclin-1 interact with each other. (**A**, **a**) Co-immunoprecipitation of p53 and Beclin-1 from wtp53 and shp53 cells by using anti-p53 antibody showing pull down of p53 and Beclin-1 from wtp53 cells (lane 1), but no p53 or Beclin-1 from shp53 cells (lane 2). (**b**) Western blots of pull downs by using anti-Beclin-1 antibody probed with anti-p53 antibody showing strong p53 immunoreactivity in wtp53 cell lysates (lane 1) and significantly lesser immunoreactivity in shp53 cell lysates (lane 2). Vps34 antibody was used as co-IP controls. (**c**) Inputs for a and b. IP, immunoprecipitation; (**B**; **a**, **b**) cytosolic (cyto) and nuclear (Nu) extracts from wtp53 cells immunoprecipitated with anti-p53 and anti-Beclin-1 antibody and immunoblotted for Beclin-1 and p53 respectively. The right panel shows the inputs as direct immunoblots of Beclin-1, p53, PARP and tubulin. IP, immunoprecipitation, IB, immunoblot. (**C**, **a**) Schematic shows domains of Beclin-1 tagged to FLAG (left panel), (**b**) Western blots of immunoprecipitates by using anti-FLAG antibody with blots probed with anti-p53 antibody. Note immunoreactivity in the region of 1–150. All western blots are representative of a minimum of 3–5 repeats.

The above experiments suggested an inverse relationship between Beclin-1 and p53 levels. This prompted us to examine interaction between the two proteins. The wtp53 and shp53 cell lysates subjected to co-immunoprecipitation (Co-IP) with anti-p53 antibody demonstrated Beclin-1 pull down with anti-p53 antibody (Fig. 2A, a, lane 1) and p53 pull down by anti-Beclin-1 antibody (Fig. 2A, b, lane 1). As a control to these the amount of p53 that could be pulled down by anti-Beclin-1 antibody from shp53 lysates was significantly less (Fig. 2A, b, lane 2). Figure 2A, c indicates input of the co-IP. The ability of both antibodies to pull down p53 and Beclin-1 together suggested an existing interaction between the two molecules. Recently, a report of p53-Beclin-1 co-immunoprecipitation under resveratrol treatment has become available; however, no cellular site of interaction, binding sites or the functional consequences of such binding were defined in this study 26. Co-IP with subcellular fractions of nucleus and the cytosol with both anti-p53 and anti-Beclin-1 antibodies showed cytosol as the primary site of interaction (Fig. 2B, a, b, lane 1) as Beclin-1 antibody could pull down p53 and p53 antibody could pull down Beclin-1 from the cytosol in detectable amounts as compared with pull downs from nuclear lysates. Figure 2B, c indicates input of the co-IP. Having established molecular association, we sought to determine the possible interaction sites on Beclin-1 for p53. Cellular extracts isolated from wtp53 cells expressing flag-tagged domains of the Beclin-1 (Bcl-2-BD-flag, CCD-flag, and ECD-flag) (Fig. 2C, a), subjected to FLAG-IP by using anti-flag antibody showed reactivity in the region of 1–150 amino acid of Beclin-1 (Fig. 2C, b). In summary, the above data demonstrated an interaction between p53 and Beclin-1 through the BH3 domain of Beclin-1 with cytosol as the primary site of interaction.

### p53 regulates Beclin-1 ubiquitination

**Figure 3:**
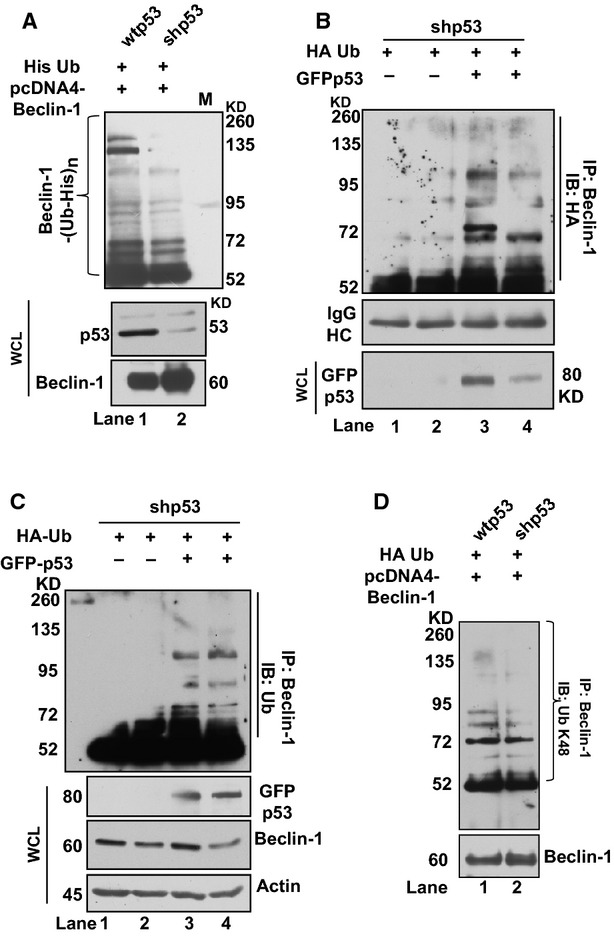
Level of Beclin-1 is maintained through ubiquitination by p53. (**A**) Western blot shows ubiquitination status of Beclin-1 in wtp53 and shp53 cells transfected with pcDNA4-Beclin-1 and His-ubiquitin (His Ub). WCL, whole cell lysate. (**B**) Western blots show effects of overexpression of p53 in shp53 cells on ubiquitination of Beclin-1. Lanes 1 and 2 are pull downs from cell lysates without p53 overexpression. Lanes 3 and 4 are pull downs from cell lysates with GFP-p53 overexpression. (**C**) Western blots show immunoprecipitates from shp53 cells transfected with GFP-p53 and HA-tagged Ub. The cell lysates immunoprecipitated with anti-Beclin-1 antibody and immune-complexes visualized with anti-ubiquitin antibody show ubiquitination after GFP-53 overexpression (lanes 3 and 4). Lanes 1 and 2 show lysates of cells that were not transfected for p53 overexpression. Whole cell lysates (WCL) were probed with anti-GFP, anti-beclin-1 and anti-actin antibody. (Ratio of Beclin-1: Actin, *lane 1*, 0.84, *lane 2*, 1.16; *lane 3*, 1.53; *lane 4*, 1.48). (**D**) wtp53 and shp53 cells transfected with cDNA constructs expressing Beclin-1 and/or HA-tagged ubiquitin probed with anti-K48 antibody. Note lesser K48 linkages formed in shp53 cells. All western blots are representative of a minimum of 3–5 repeats.

Protein–protein interactions may initiate processes like ubiquitination leading to protein degradation for cellular quality control 27. Prior knowledge shows that ubiquitination is important for Beclin-1 both for its regulation as well as for its degradation 18. Therefore, to evaluate if Beclin-1 ubiquitination was occurring, proteasome inhibitor MG132 was used and the levels of both p53 and Beclin-1 were followed. In both the sc shRNA transfected wtp53 cells and shp53 cells, accumulation of Beclin-1 occurred till 10 hrs after which it declined (Fig. S2A). The accumulation in the presence of a proteasome inhibitor suggested ubiquitin-mediated degradation of Beclin-1. We transfected both wtp53 and shp53 cells simultaneously with constructs expressing His-tagged ubiquitin and Beclin-1. His-purified proteins from extracts of these cells probed with anti-Beclin-1 antibody showed lesser ubiquitination of Beclin-1 in shp53 cells (Fig. 3A, lane 2) as compared with wtp53 cells (Fig. 3A, lane 1). Arguably, if lesser p53 was responsible for reduced ubiquitination of Beclin-1, overexpression of p53 should restore normal levels of Beclin-1 ubiquitination. The shp53 cells concurrently transfected with GFP-p53 constructs to compensate for the low p53 and a plasmid containing a HA-tagged ubiquitin protein (HA-Ub) showed higher Beclin-1 ubiquitination in shp53 cells (Fig. 3B, lanes 3–4) as compared with cells with vector-only control (Fig. 3B, lanes 1–2). The same blot probed with anti-ubiquitin antibody showed similar results of higher Beclin-1 ubiquitination in the presence of GFP-p53 (Fig. 3C). Levels of GFPp53, Beclin-1 and actin are shown below. The ratio of Beclin-1:actin is shown in the figure legends. Therefore, the above experiments clearly showed that p53 levels were important for Beclin-1 ubiquitination, where low p53 was favourable for decreased ubiquitination of Beclin-1. As ubiquitination occurs at specific lysine residues, we tested if Beclin-1 ubiquitination was K48-linked because K-48 linked ubiquitination is related to proteasome-mediated degradation 12. IP of Beclin-1 under denaturing conditions, followed by immunoblot with K48-linkage specific anti-ubiquitin antibodies showed lesser ubiquitination at K48 in shp53 cells (Fig. 3D, lane 2) as compared with the wtp53 cells (Fig. 3D, lane 1). The above observations support the hypothesis that p53 is necessary for Beclin-1 ubiquitination and in the absence of p53, Beclin-1 accumulates.

### Beclin-1–p53 interaction is disrupted by cisplatin exposure

**Figure 4:**
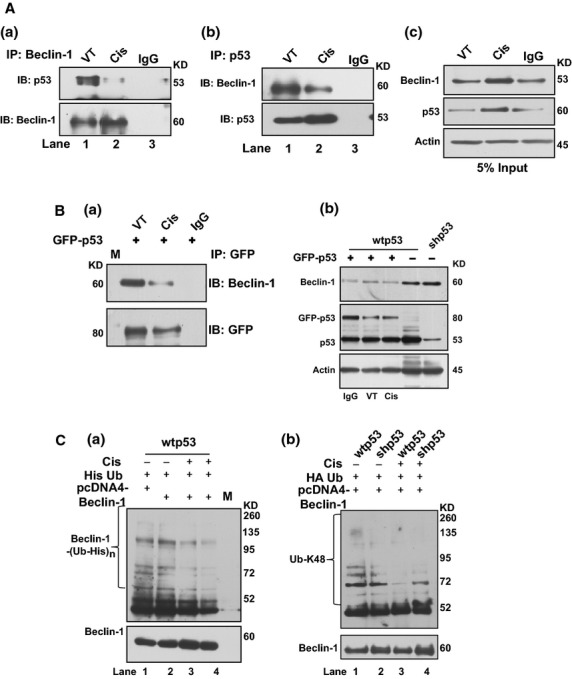
Cisplatin reduces p53–Beclin-1 interaction. (**A**; **a**, **b**) Co-immunoprecipitation assays for the endogenous interaction between p53 and Beclin-1 show loss of interaction upon cisplatin (1 μg/ml) treatment as visible in immunoprecipitates with anti-Beclin-1 (**a**) and anti-p53 antibody (**b**) probed with anti-p53 antibody (**a**) and anti-Beclin-1 antibody (**b**). (**c**) It shows the input. IP, immunoprecipitation; IB, immunoblot. (**B**, **a**) Immunoprecipitation data from GFP-p53 transfected cells with anti-GFP antibody probed with anti-Beclin-1 antibody showing reduction of p53–Beclin-1 interaction post cisplatin treatment. IP, immunoprecipitation; IB, immunoblot. (**b**) Western blots showing decrease in Beclin-1 levels in GFP-53 overexpressed cells in comparison to wtp53 cells without overexpression. (**C**, **a**) Western blot shows ubiquitination status of Beclin-1 in the presence of cisplatin in wtp53 cells where His-ubiquitin and Beclin-1 expression vectors were simultaneously co-expressed prior to treatment with cisplatin (1 μg/ml) for 6 (lane 3) and 12 hrs (lane 4). Nickel-agarose purified proteins probed with anti-Beclin-1 antibody show Beclin-1 immunoreactivity in the Beclin-1 blot. (**b**) Cell lysates treated with cisplatin (1 μg/ml) immunoprecipitated with anti-Beclin-1 antibody probed with Ub-K48 antibody show lesser linkages formed after cisplatin treatment (lanes 3–4) as compared with untreated cells (lanes 1–2). All western blots are representative of a minimum of 3–4 repeats.

To establish the importance of p53–Beclin-1 interaction, we investigated if stress had any influence on the interaction as that could potentially alter the results of treatment. We used cisplatin, a platinum-containing anti-cancer drug 27 to which EC cells are sensitive 21, to determine whether the status of Beclin-1–p53 interaction changes in response to drug-induced stress and if so the possible biological consequences of such changes. A dose–response curve with different doses of cisplatin showed an increase in Beclin-1 upon cisplatin treatment till the dose of 2 μg/ml in wtp53 cells, after which Beclin-1 levels declined at higher dosages (data on request). Cisplatin dose of 1 μg/ml was chosen for further investigations based on the highest cellular Beclin-1 expression induced by that dose. To see if cisplatin stress influenced the molecular interaction between p53 and Beclin-1, co-IPs were conducted with Beclin-1 and p53 antibodies with lysates made from cisplatin-treated wtp53 cells. A distinct reduction in the amount of immunoprecipitable p53 with anti-Beclin-1 antibody was observed in comparison to vehicle-treated cells (Fig. 4A, a, lanes 2 and 1). Similarly, co-IP with anti-p53 antibodies could pull down less Beclin-1 from lysates of cisplatin-treated cells rather than untreated cells (Fig. 4A, b, lane 2 and 1). As a further control, we compensated p53 by enforced expression of GFP-p53 in shp53 cells and subsequently pulled down the proteins (with anti-GFP antibodies) from cell lysates exposed to cisplatin. The lysates from cisplatin-treated cells showed lesser Beclin-1 pull down as compared with only vehicle-treated cells (Fig. 4B, a). Figure 4B, b indicates input of the co-IP. The reduced pull down of Beclin-1 after cisplatin treatment was indicative of decreased interaction of Beclin-1 and p53. Therefore, to identify if this reduced interaction changed the status of Beclin-1 ubiquitination, wtp53 cells were simultaneously transfected with Beclin-1 as well as His-Ub expression vectors and exposed to cisplatin. His-purified extracts from these cells with and without treatment with cisplatin showed lesser ubiquitination of Beclin-1 in treated cells (Fig. 4C, a, lanes 3–4) as compared with untreated cells (Fig. 4C, a, lanes 1–2). These data supported the idea that cisplatin treatment reduced Beclin-1 ubiquitination. Expectedly, formation of K48 linked ubiquitin chains was lesser in cells treated with cisplatin (Fig. 4C, b, lanes 3–4) as compared with untreated cells (Fig. 4C, b, lanes 1–2). The above studies clearly demonstrated that the interaction of p53–Beclin-1 was disrupted during cisplatin treatment resulting in lesser ubiquitination of Beclin-1.

### Disruption of Beclin-1–p53 interaction increases autophagy and inhibition of autophagy promotes cell death

**Figure 5:**
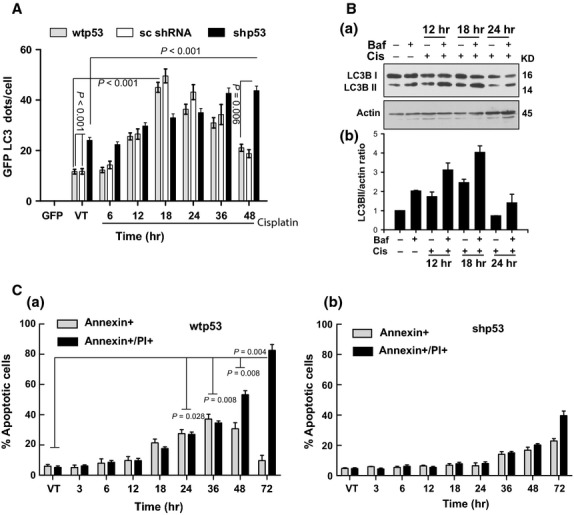
Inhibition of autophagy increases cell death. (**A**) Quantification of GFP-LC3 puncta per cell in wtp53, sc shRNA transfected and shp53 cells after cisplatin (1 μg/ml) treatment (0–48 hrs). GFP-LC3 dots in each cell (100) were counted in at least five independent visual fields. Bar graph shows mean ± SEM of 3 independent experiments. (**B**) Wtp53 cells were treated with cisplatin (1 μg/ml) as per indicated time followed by 100 nM bafilomycin A1 during the last 4 hrs (**a**) and LC3BII was quantified by densitometry analysis normalized to actin (**b**). Western blot is representative of a minimum of 3–5 repeats. (**C**) Annexin-V/PI assay after cisplatin treatment at different time-points, (**a**) wtp53 cells; (**b**) shp53 cells. Note lower no. of annexin-V+ve shp53 cells. Data represent mean ± SEM of 3 independent experiments.

To look at the biological consequences of alterations in p53 and Beclin-1 interaction, the extent of cellular autophagy and apoptosis was determined. When autophagy was measured after cisplatin treatment, in wtp53- and sc shRNA treated cells, autophagy increased till 18 hrs after which the readings showed a decline (Fig. 5A). In contrast, the shp53 cells with higher constitutive autophagy did not show as high an increase at earlier time-points, but increased later (Fig. 5A). The autophagy flux experiments performed with bafilomycin A1 showed highest LC3BII at 18 hrs post cisplatin in wtp53-treated cells (Fig. 5B) confirming the microscopy data shown in Figure 5A. As shown in Figure 5C, a, cell death in wtp53 cells started increasing after the autophagic activity started going down after 18 hrs suggesting a protective action of autophagy. In contrast, shp53 cells where autophagy was constitutively high showed no significant increase in cell death till 48 hrs (Fig. 5C, b). Therefore, the above experiments with both wtp53 and shp53 cells showed that higher autophagy was linked to less cell death 28.

**Figure 6:**
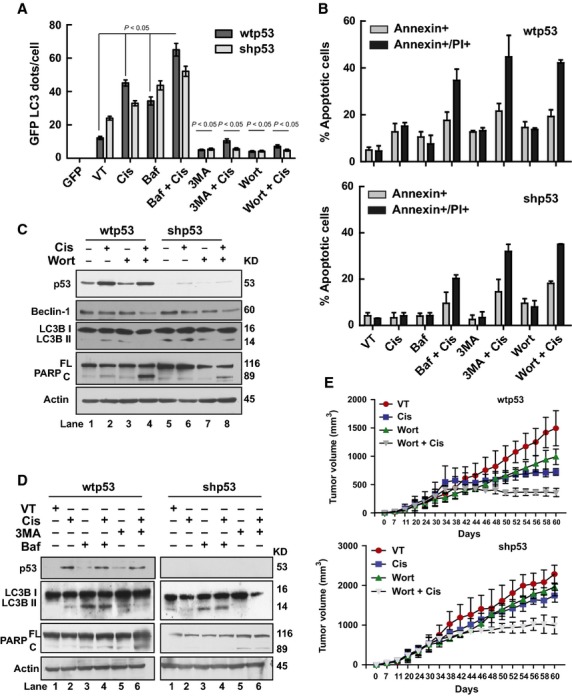
Inhibition of autophagy enhances cisplatin-induced apoptosis. (**A**) Bar graph showing counts of GFP-LC3 puncta in GFP-LC3 transfected wtp53 and shp53 cells, incubated with autophagy inhibitors [bafilomycin A1 (10 nM), 3MA (5 mM) and wortmannin (100 nM)] prior to cisplatin treatment. Note accumulation of autophagosomes with bafilomycin A1 and reduction of LC3 puncta with other inhibitors. The results are mean ± SEM of 3 independent experiments; 50–100 cells were analysed per assay. (**B**) Annexin-V/PI labelling analysis of wtp53 and shp53 cells treated with cisplatin (1 μg/ml) in the presence or absence of autophagy inhibitors showing higher apoptosis in inhibitor-treated groups. Annexin, annexin-V; PI, propidium iodide. (**C**) Autophagy inhibitor wortmannin (100 nM) was added to wtp53 and shp53 cells during cisplatin (1 μg/ml) treatment. Western blots show levels of various proteins from these lysates. Actin was used as a loading control. (**D**) Autophagy inhibitors 3-MA (5 mM) and bafilomycin A1 (10 nM) were added to wtp53 and shp53 cells during cisplatin (1 μg/ml) treatment. Western blots show levels of p53, LC3BII and cleaved PARP protein from these lysates. All western blots are representative of a minimum of 3–5 repeats. (**E**) Athymic nude mice (*nu/nu*) harbouring small established subcutaneous xenografts of human wtp53 and shp53 human EC cells treated with cisplatin in the presence of wortmannin show tumour sizes after various treatments. Note that tumour volume is significantly less in the combination group in comparison to monotherapy. Data are mean ± SEM (*n* = 4).

Arguably, if higher autophagy was linked to less cell death, inhibition of autophagy should remove the protective action and increase cell death. Therefore, we proceeded to carry out experiments with autophagy inhibitors like bafilomycin A1, a specific inhibitor of vacuolar H^+^ ATPase, 3MA and wortmannin, both selective inhibitors of phosphatidylinositol 3-kinase to see the effects of autophagy inhibition on the outcome of cisplatin treatment. All three inhibitors reduced autophagy that was visible in reduced LC3 puncta with 3MA and wortmannin (Fig. 6A). Bafilomycin A1 showed high LC3 puncta because it blocks autophagy at a later stage by inhibiting the fusion between autophagosomes and lysosomes (Fig. 6A) 29. As shown in Figure 6B, autophagy inhibitors increased cisplatin-induced cell death both in wtp53 and shp53 cells. Figure 6C shows PARP cleavage (Fig. 6C, lanes 4 and 8) indicative of apoptosis that corroborated the annexin-PI data shown in Figure 6B. In addition to pharmacological inhibition, we used shRNA-mediated down-regulation of Beclin-1 and ATG-5 to reduce autophagy and exposed the cells to cisplatin. Two shRNAs Beclin-1 29 and 32 and A-33 and A-36 of ATG-5 were able to reduce Beclin-1 and ATG-5 levels, respectively, and were used along with the drug. Figure S3 shows that when cisplatin was given to wtp53 and shp53 cells in Beclin-1 down-regulated conditions, PARP cleavage occurred (Fig. S3B) suggesting apoptotic cell death in these groups as compared with sc shRNA transfected cells treated with cisplatin. This was corroborated by Annexin-PI staining (Fig. S3C) clearly showing that cisplatin is more effective under autophagy down-regulated conditions.

To see if autophagy inhibition had similar effects *in vivo*, xenograft tumours formed by both wtp53 and shp53 cells were treated with cisplatin in the presence of wortmannin. A significant decline in tumour volume was recorded when cisplatin treatment was given in the presence of wortmannin (Fig. 6E). This confirmed cisplatin was more efficient during autophagy inhibited conditions. Thus, data from *in vitro* experiments and *in vivo* xenograft tumours confirm that autophagy inhibition increased cisplatin efficacy.

## Discussion

Combined effects of apoptosis and autophagy in the determination of cell fate are well recognized and much interest is generated in targeting the autophagy pathways for cancer drug discovery 30. As alterations in the kinetics of autophagy and apoptosis have a potential therapeutic application 30, it is important to understand the processes in a variety of tumour cells. Our earlier data with EC cells showed increased cell survival upon down-regulation of p53 21; however, the mechanism by which p53 was regulating cell survival was not understood. This report provides a physiological mechanism for autophagy regulation in EC cells through a novel observation that p53 and Beclin-1 interacting through the Beclin-1 BH3 domain determine autophagy status of cells. In addition, it shows that changes in autophagic activity upon drug treatment lead to alterations in the kinetics of apoptosis, thus changing outcome of the treatment. The consequences of Beclin-1–p53 interaction mediated through the BH3 domain of Beclin-1 are important because the Bcl-2 family members bind to Beclin-1 through this domain 31 and competition with p53 may have impact on the determination of cell fate. However, these EC cells do not express Bcl-2 32; therefore, such interaction is not possible, although interaction with other Bcl-2 family members may occur. Our observation of reduced autophagy when p53 and Beclin-1 are interacting is similar to observations with Bcl-2 and Beclin-1 interactions reported in other cell types 33.

For this study, the p53 down-regulated cells were ideal to experimentally provide evidence of Beclin-1 behaviour in the presence or absence of p53. EC cells typically express very high levels of p53 and are therefore able to withstand overexpressed p53 proteins without death signals being activated 34. Prior evidence links high Beclin-1 to increased autophagy with two possible outcomes of either favouring cell survival or precipitating death after a threshold of autophagic activity is crossed 35. It is evident from our studies that p53 down-regulation was causal to Beclin-1 increase, primarily as a result of decreased ubiquitination of Beclin-1. Beclin-1 ubiquitination is a known physiologically relevant event in tuning the function of the protein through proteolytic and non-proteolytic means 12, but its association with p53 for ubiquitination is not known. Regulation of p53 levels by Beclin-1 through USP10-mediated deubiquitination has been reported 36, but p53-mediated regulation of Beclin-1 has not been described. This study establishes the requirement of p53 for Beclin-1 ubiquitination in EC cells. The K48-mediated Beclin-1 ubiquitination under non-stressed conditions observed in this study agrees with reports of formation of K48-linked ubiquitin chains on Beclin-1 in monocytes by HSP90 12.It is pertinent to mention here that Beclin-1 can also be targeted by NEDD4, which interacts with Beclin-1 37; however, we have not investigated NEDD4-mediated Beclin-1 ubiquitination in EC cells.

Importantly, our observations indicate a possible reason for relative cisplatin inefficacy because the drug induces dissociation of p53 Beclin-1 resulting in higher autophagy and reduced cell death. The causality of autophagy changes to inhibition of cell death was established by observations of elevation of cell death levels when autophagy was blocked during drug treatment through pharmacological or genetic means. The *in vitro* observations were verified by *in vivo* data from xenograft tumours formed in *nu/nu* mice, where cisplatin treatment in the presence of an autophagy inhibitor wortmannin favoured reduction of tumour size faster than by cisplatin-only treatment. These data support observations on the refractoriness of tumours to treatment when autophagy increases 38,39. This is a paradoxical situation because autophagy can also help in tumour regression as opposed to being protective 39. It is of interest that p53 down-regulated cells expressing higher autophagy formed tumours faster than wild-type cells, again suggesting a tumour-promoting effect of autophagy.

Mechanistic overlap between apoptosis and autophagy is known to involve a variety of proteins 33, and in this context the p53–Beclin-1 interaction may be yet another interaction to serve as a key regulatory element in controlling autophagy. Like many protein–protein interactions that may be interfered with, the p53–Beclin-1 interaction could be maintained to prevent autophagy from increasing during drug treatment. The protective nature of autophagy in the EC cells suggests that treatment of EC tumours could improve if chemotherapy is combined with autophagy inhibitors as our mice xenograft studies suggest. In a clinical setting, there is a great interest in manipulating autophagy to generate more effective drug treatment 39. Clinical trials are ongoing where autophagy inhibition has been combined with chemotherapy under the premise that damaged cells can die without being rescued by the process of autophagy in a variety of tumours like glioblastoma, B-cell chronic lymphocytic leukaemia, breast cancer and lung cancer, but not with EC 40,41. Therefore, our studies contribute findings towards the ongoing dialogue on the utility of autophagy manipulation for improvement of therapeutic strategy to enhance the efficacy of anti-cancer therapies.

## Supporting Information

**Figure S1** (A) Down-regulation of p53 by p53 shRNA (lane 3) as compared to sc shRNA transfected cells (lane 2) or untransfected wtp53 cells (lane 1). (B) Tissue sections from the tumours stained for p53, LC3BII and Beclin-1. Note low p53 correlates to high LC3BII and Beclin-1 stain indicating higher autophagy.

**Figure S2** A. The sc shRNA transfected wtp53 cells and shp53 cells were treated with 5 μM MG132, the proteasome inhibitor along with cisplatin.

**Figure S3** (A) Level of Beclin-1 and ATG-5 in cell lysates of wtp53 cells transfected with Beclin-1 and ATG-5 shRNA for 48 hrs. Note the efficacy of only sh B29 and 32 in reducing Beclin-1 levels. (B) Western blots show the effect of down-regulation of Beclin-1 on PARP cleavage and LC3BII formation. (C) Data showing increased apoptosis in cells transfected with sh B29 and sh B32 (shRNA against Beclin-1) and sh A33 and A36 (shRNA against ATG5).

**Table S1** List of constructs used in the study.
