# Supplementary material for: Beclin-1–p53 interaction is crucial for cell fate determination in embryonal carcinoma cells
Source: J Cell Mol Med. 2014 Sep 11;18(11):2275–86. doi: 10.1111/jcmm.12386 (PMC4224560; doi:10.1111/jcmm.12386)
Supplement: Supplementary file 4 — Table S1 List of constructs used in the study. [file jcmm0018-2275-sd4.docx]

**Table 1. List of constructs used in the study**

| **Vector** | **Company** | **Annotation** |
| --- | --- | --- |
| shp53 pLKO.1 puro | Addgene, Cambridge, USA | Addgene Plasmid 19119 from Bob Weinberg (1) |
| EGFP-LC3 | Addgene, Cambridge, USA | Addgene Plasmid 11546 from Karla Kirkegaard (2) |
| GFP-p53 | Addgene, Cambridge, USA | Addgene Plasmid 12091 from Tyler Jacks  (3) |
| pcDNA3-Beclin-1 | Addgene, Cambridge, USA | Addgene Plasmid 21150 from Junying Yuan (4) |
| pBABEpuro GFP-LC3 | Addgene, Cambridge,USA | Addgene Plasmid 22405 from JayantaDebnath (5) |
| pMD2.G | Addgene, Cambridge, USA | Addgene Plasmid 12259 from Didier Trono |
| Scramble shRNA | Addgene, Cambridge, USA | Addgene Plasmid 1864 from David M. Sabatini (6) |
| psPAX2 | Addgene, Cambridge, USA | Addgene Plasmid 12260 from Didier Trono |
| pcDNA4-Beclin-1 1-150 | Addgene, Cambridge, USA | Addgene Plasmid 24389 from Qing Zhong (7) |
| pcDNA4-Beclin-1 151-241 | Addgene, Cambridge, USA | Addgene Plasmid 24390 from Qing Zhong (7) |
| pcDNA4-Beclin-1 243-450 | Addgene, Cambridge, USA | Addgene Plasmid 24392 from Qing Zhong (7) |
| pEGFP N1 | Clontech Laboratories, Inc. CA,USA |  |
| pcDNA^TM^ 3.1 | Invitrogen, Eugene, OR |  |
| pCI-His-hUbi | Addgene, Cambridge, USA | Addgene Plasmid 31815 from Astar Winoto (8) |
| HA-Ubiquitin | Addgene, Cambridge, USA | Addgene Plasmid 18712 from Edward Yeh (9) |

All the constructs were sequenced for validation prior to use.

**References**

1. Godar, S., Ince, T. A., Bell, G. W., Feldser, D., Donaher, J. L., Bergh, J., Liu, A., Miu, K., Watnick, R. S., Reinhardt, F., McAllister, S. S., Jacks, T., and Weinberg, R. A. (2008) Growth-inhibitory and tumor- suppressive functions of p53 depend on its repression of CD44 expression. *Cell* **134,** 62-73

2. Jackson, W. T., Giddings, T. H., Jr., Taylor, M. P., Mulinyawe, S., Rabinovitch, M., Kopito, R. R., and Kirkegaard, K. (2005). Subversion of cellular autophagosomal machinery by RNA viruses. *PLoS. Biol.* **3,** e156

3. Boyd, S. D., Tsai, K. Y., and Jacks, T. (2000) An intact HDM2 RING-finger domain is required for nuclear exclusion of p53. *Nat. Cell Biol.* **2,** 563-568

4. Shibata, M., Lu, T., Furuya, T., Degterev, A., Mizushima, N., Yoshimori, T., MacDonald, M., Yankner, B., and Yuan, J. (2006) Regulation of intracellular accumulation of mutant Huntingtin by Beclin 1. *J. Biol. Chem.* **281,** 14474-14485

5. Fung, C., Lock, R., Gao, S., Salas, E., and Debnath, J. (2008) Induction of autophagy during extracellular matrix detachment promotes cell survival. *Mol. Biol. Cell* **19,** 797-806

6. Sarbassov, D. D., Guertin, D. A., Ali, S. M., and Sabatini, D. M. (2005) Phosphorylation and regulation of Akt/PKB by the rictor-mTOR complex. *Science* **307,** 1098-1101

7. Sun, Q., Fan, W., Chen, K., Ding, X., Chen, S., and Zhong, Q. (2008) Identification of Barkor as a mammalian autophagy-specific factor for Beclin 1 and class III phosphatidylinositol 3-kinase. *Proc. Natl. Acad. Sci. U. S. A* **105,** 19211-19216

8. Young, J. A., Sermwittayawong, D., Kim, H. J., Nandu, S., An, N., Erdjument-Bromage, H., Tempst, P., Coscoy, L., and Winoto, A. (2011) Fas-associated death domain (FADD) and the E3 ubiquitin-protein ligase TRIM21 interact to negatively regulate virus-induced interferon production. *J. Biol. Chem.* **286,** 6521-6531

9. Kamitani, T., Kito, K., Nguyen, H. P., and Yeh, E. T. (1997) Characterization of NEDD8, a developmentally down-regulated ubiquitin-like protein. *J. Biol. Chem.* **272,** 28557-28562
